# Relational and contextual dynamics in online teaching Turkish as a foreign language: toward a relationally mediated learning system

**DOI:** 10.3389/fpsyg.2026.1888441

**Published:** 2026-07-13

**Authors:** Bilge Nur Dogan Guldenoglu

**Affiliations:** Police Academy, Ankara, Türkiye

**Keywords:** heritage language, online language teaching, relationally mediated learning, second language learning (L2), transnational contexts

## Abstract

This study offers a reconceptualization of online second and heritage language learning as a relationally mediated and context-dependent process, thereby addressing a key limitation in existing individualistic and technologically oriented models, which tend to treat relational dynamics as secondary rather than constitutive elements of learning. Drawing on a multi-stakeholder qualitative design, it examines how students, parents, and teachers conceptualize and experience learning Turkish in transnational online contexts, where Turkish functions as both a second/foreign language and a heritage language depending on learners' linguistic and family backgrounds. Data were collected through semi-structured interviews with 27 participants and analyzed using thematic analysis. Findings indicate that language learning is primarily grounded in relational and identity-based meanings, with family interaction and cultural continuity serving as central drivers of engagement. Beyond meaning construction, learning emerges as a dynamic system shaped by the interaction of learner characteristics, instructional mediation, and contextual conditions, with relational dynamics regulating how these elements are experienced. Online instruction is not perceived as a neutral technological medium, but as an interactionally constructed environment in which engagement and relational connection shape participation. Building on these findings, the study advances a relationally mediated learning system that integrates meaning, learning processes, and engagement within a unified framework. By foregrounding relational mechanisms as constitutive elements of learning, it offers a theoretically robust and context-sensitive account of language learning in complex, transnational, and digitally mediated environments.

## Introduction

Language learning in online and transnational contexts, particularly in the domain of second and foreign language (L2) and heritage language learning, is often conceptualized as an individual, skill-based process driven by cognitive development, instructional input, and technological affordances (Gass et al., 2025). Within this perspective, learning outcomes are typically explained in terms of learners' abilities, the effectiveness of instructional design, and the features of digital environments that enable or constrain participation (Hiver et al., 2021; Lai, 2019). Such approaches have contributed significantly to understanding second language acquisition, particularly by highlighting the roles of motivation, input, interaction, and learner engagement in shaping learning processes (Boo et al., 2015; Kim, 2023; Sun et al., 2025, 2026a,b). Recent studies of AI-mediated and informal digital language learning further suggest that learners' beliefs, self-guides, achievement emotions, and patterns of engagement shape how digital environments are experienced and utilized for language development (Wang et al., 2025, 2026; Wang and Wang, 2024). Taken together, these theoretical and empirical traditions—including cognitive and skill-based approaches to second language acquisition, technology-mediated perspectives on digital language learning, and engagement-oriented models of online instruction—have substantially advanced understanding of language learning processes (Gass et al., 2025; Hiver et al., 2021; Lai, 2019). However, they have predominantly examined cognitive, instructional, technological, or motivational dimensions, with comparatively less attention to the relational mechanisms through which learners, families, and teachers jointly construct and sustain language learning experiences. However, by privileging individual and instrumental dimensions, these perspectives provide an incomplete account of how language learning is experienced, interpreted, and sustained within socially embedded and context-dependent systems (Kim, 2020; Larsen-Freeman, 2018). Although these perspectives have generated important insights into digital language learning, they provide comparatively less attention to the relational and socially embedded mechanisms through which engagement and participation are co-constructed across learners, families, and teachers. These considerations are particularly relevant to online Turkish language learning in transnational contexts, where language development is closely intertwined with family relationships, heritage maintenance, and identity construction.

Although research on online and heritage language learning has expanded, it remains fragmented across cognitive, instructional, and technological perspectives (Curle et al., 2020; Macaro, 2022; Macaro et al., 2018; Rose et al., 2026). This fragmentation is also reflected in broader educational research, where growing attention to teacher-related factors has highlighted the need for more integrated and context-sensitive conceptual frameworks that connect instructional, relational, and learner-centered dimensions of teaching and learning (Wang et al., 2024). Studies on heritage language learning emphasize family practices and intergenerational transmission, while online learning research focuses on engagement, interaction, and digital affordances (Curdt-Christiansen et al., 2023; Li et al., 2026). However, these strands are rarely integrated within a unified framework, and relational processes are often treated as secondary rather than central mechanisms of learning (Kusyk et al., 2025), leaving important questions about how relational, instructional, and engagement processes interact to shape language learning insufficiently understood. As a result, existing models fail to explain how meaning, engagement, and learning processes are co-constructed within socially embedded and context-dependent systems, particularly in transnational environments shaped by family dynamics and identity negotiation (Leung and Valdes, 2019; Wei, 2018).

Rather than replacing these established perspectives, the present study seeks to complement them by foregrounding relational processes as constitutive elements through which meaning, engagement, and learning are co-constructed in online and transnational contexts (Hiver et al., 2021). Without such a perspective, engagement, difficulty, and instruction risk being misinterpreted as individual or technical phenomena rather than as components of a dynamically interacting system (Dunn and Iwaniec, 2022; Macaro, 2020; Zhou and Thompson, 2023). Accordingly, the present study repositions relational processes as the primary organizing principle of language learning.

An important conceptual consideration in the present study concerns the relationship among second/foreign language (L2), Turkish language learning, and heritage language learning. In transnational contexts, these categories are not always mutually exclusive. While some learners study Turkish primarily as an additional language with limited prior exposure, others encounter it as a heritage language connected to family practices, cultural continuity, and identity. Moreover, individual learners may simultaneously occupy both positions, receiving formal L2 instruction while also experiencing varying degrees of informal exposure within the home environment. Rather than treating these categories as strictly separate, the present study adopts a broader perspective of online Turkish language learning in transnational settings, encompassing both L2 and heritage language experiences and focusing on the relational and contextual processes that shape learning across these contexts.

This need becomes particularly salient in the context of teaching Turkish as a foreign (L2) or heritage language in transnational settings, where learning is closely intertwined with family relationships, cultural continuity, and identity construction (Curdt-Christiansen et al., 2023; Li et al., 2026). Unlike dominant global languages often learned for academic or economic purposes, Turkish in diaspora contexts is typically maintained as a heritage language embedded in everyday family interaction and emotionally meaningful communication (Coskun Kunduz, 2022). Opportunities for language use are therefore largely confined to home environments and interactions with extended family, resulting in a learning process that is both context-bound and relationally driven (Bayram and Wright, 2018).

At the same time, the shift toward online instruction introduces additional complexity, as learning occurs in digitally mediated environments where interaction and engagement must be actively constructed (Bolliger and Martin, 2018; Hodges et al., 2020). Despite these characteristics, existing research remains insufficient to explain how relational, instructional, and contextual dimensions intersect in shaping language learning in online and transnational contexts. This gap calls for a system-level perspective that conceptualizes learning as an emergent process arising from their interaction.

Addressing this gap, the present study adopts a multi-stakeholder qualitative approach to examine how students, parents, and teachers experience learning Turkish as a foreign (L2) language in online contexts. It focuses on how meaning construction, learning processes, and engagement are shaped through relational and context-dependent dynamics. By integrating stakeholder perspectives, the study advances a *relationally mediated learning system* that conceptualizes language learning as an emergent process arising from the interaction of meaning, instructional mediation, and engagement.

Within this framework, these dimensions are treated as mutually constitutive elements of a unified system, offering a new lens for understanding how learning is experienced and sustained in online and transnational contexts. By explicitly foregrounding relational mechanisms, the study contributes to language learning research by challenging individualistic and technologically deterministic perspectives and by positioning learning as a dynamically regulated and socially grounded process.

### The present study

This study examines how students, parents, and teachers conceptualize and experience online Turkish language learning in transnational contexts, where Turkish may function as both an L2/foreign language and a heritage language. Adopting a multi-stakeholder qualitative design, the study moves beyond descriptive accounts of learning experiences to investigate how meaning, engagement, and instructional processes are relationally constructed and dynamically interconnected.

Specifically, the study conceptualizes online Turkish language learning as a relationally mediated system comprising three interconnected dimensions. First, it explores how stakeholders construct the meanings and purposes of learning Turkish, providing the motivational and relational foundations of participation. Second, it examines the learner-related, instructional, and contextual factors that shape the learning process itself. Third, it investigates how these dynamics are experienced within online instructional environments through patterns of engagement, interaction, and relational connection. Rather than treating these dimensions as independent phenomena, the study views them as complementary components of a unified learning system and examines both shared and stakeholder-specific perspectives across students, parents, and teachers.

The following research questions guide the study. The three research questions are intentionally sequential and complementary. They move from stakeholders' constructions of the meaning and purposes of learning Turkish to the factors shaping the learning process, and finally to the ways these processes are experienced within online instructional environments. Together, they provide an integrated understanding of online Turkish language learning as a relationally mediated system.

RQ1. How do students, parents, and teachers construct the meanings and purposes of learning Turkish in transnational online contexts?

RQ2. How do students, parents, and teachers perceive the learner-related, instructional, and contextual factors shaping online Turkish language learning?

RQ3. How do students, parents, and teachers experience online Turkish language instruction in terms of engagement, interaction, and relational dynamics?

## Method

### Research design

This study was designed as a multi-perspective qualitative study grounded in an interpretive framework to explore how different stakeholders conceptualize and experience learning and teaching Turkish in online transnational contexts. Given the study's aim to capture multiple perspectives and meaning-making processes, a multi-stakeholder approach was adopted, incorporating the views of students, parents, and teachers. This design enabled a comprehensive examination of both shared and stakeholder- specific experiences, as well as the relational and contextual dynamics shaping online language learning. The study was guided by a thematic analysis framework, allowing for the systematic identification and interpretation of patterns across participants' accounts.

### Participants

The study included three stakeholder groups involved in online Turkish as a foreign language instruction: students (*n* = 10), their parents (*n* = 10), and teachers (*n* = 7). This multi-stakeholder composition was purposefully designed to capture complementary perspectives on the meanings, processes, and experiences of learning Turkish in online contexts (Table 1).

**Table 1:** Participant characteristics.

Stakeholder	*n*	Key characteristics
Students	10	Aged 10–14; enrolled in regular online Turkish courses; living in transnational contexts; varying levels of home exposure to Turkish
Parents	10	Living in Belgium, France, Germany, UK, Switzerland, USA, and China; diverse professional backgrounds; varying home language practices
Teachers	7	Female; minimum 5 years of experience; experience teaching Turkish to international learners in online settings

Student-parent pairs were matched to capture complementary perspectives within the same learning context.

Participants were recruited using purposive sampling to ensure the inclusion of individuals with direct experience in online Turkish language instruction. Selection criteria included active participation in online Turkish courses (for students and parents) and professional experience in teaching Turkish as a foreign language in online settings (for teachers). The inclusion of matched student–parent pairs enabled the examination of parallel perspectives within the same learning context, allowing for a more nuanced analysis of how experiences and meanings are constructed across stakeholders. All participants were informed about the purpose of the study, and informed consent was obtained prior to data collection. Participation was voluntary, and all responses were anonymized to ensure confidentiality.

Students ranged in age from 10 to 14 years and were enrolled in regular individual online Turkish language courses, typically receiving 1 h of instruction per week. All were living in transnational contexts and had varying levels of exposure to Turkish within the home environment, reflecting diverse patterns of heritage language use and maintenance.

Parents represented a highly educated and internationally distributed group residing across several countries in Europe, North America, and Asia. Most parents had lived abroad for extended periods (ranging from 4 to over 40 years) and reported that their children were primarily raised outside Türkiye. While some families maintained consistent Turkish language use at home, others reported limited use, indicating variability in linguistic environments. Parents' educational backgrounds ranged from undergraduate to postgraduate levels, and their professional profiles included fields such as law, engineering, health, finance, and architecture.

Teachers (*n* = 7) were all female and had a minimum of 5 years of professional experience teaching Turkish in online international contexts. Their experience with learners from diverse linguistic and cultural backgrounds enabled them to provide informed perspectives on online Turkish language instruction.

Overall, the participant group represents a diverse and ecologically valid sample of stakeholders engaged in online Turkish language education in transnational contexts, supporting the examination of shared and stakeholder-specific patterns in meaning construction, instructional processes, and learning experiences. The adequacy of the sample size was evaluated using the concept of information power (Malterud et al., 2016), which suggests that smaller samples are sufficient when data are rich and relevant to the research aim. In this study, high information power was supported by the specificity of the research focus, the inclusion of multiple stakeholder groups, and the depth of the qualitative data. The consistency of patterns across participants further indicated sufficient informational depth. Accordingly, the sample size was considered adequate for addressing the research questions and enabling meaningful thematic analysis.

### Data collection

Data were collected through individual semi-structured interviews conducted between September–December 2025 via online video conferencing platforms (e.g., Zoom). The interviews were guided by open-ended questions designed to elicit detailed, context-rich, and participant-driven responses. Separate interview protocols were developed for each stakeholder group (students, parents, and teachers). The protocols were structured around the three main dimensions of the study, with five questions addressing meaning construction (Theme 1), four questions focusing on factors shaping the learning process (Theme 2), and three questions examining experiences of online instruction (Theme 3).

While the thematic structure of the interview protocols was consistent across stakeholder groups, the wording of the questions was adapted to ensure clarity and relevance for each group. During the interviews, follow-up prompts were used when necessary to clarify and deepen participants' responses, allowing for a more nuanced exploration of their perspectives.

Following the initial development, the interview protocols were reviewed by an expert in teaching Turkish as a foreign language and two experienced teachers in the field. Feedback focused on content coverage and clarity of expression, and minor revisions were made to enhance the comprehensibility and relevance of the questions. The protocols were also piloted with a small group of participants, and minor adjustments were made to improve clarity and flow. In addition, selected questions invited participants to use metaphors to describe their experiences, enabling the exploration of deeper conceptualizations.

All interviews were conducted individually in an online setting and were audio-recorded with participants' consent. Given differences in language proficiency and age, student interviews were conducted with parental support when necessary to facilitate communication, while care was taken to ensure that students' responses reflected their own perspectives. The recordings were subsequently transcribed verbatim by the research team and compiled into a single dataset for qualitative analysis. To ensure accuracy, the transcripts were checked against the original audio recordings and corrected where necessary before analysis. During transcription, all identifying information was removed, and participants were assigned identification codes (e.g., T1, S1, and P1) to ensure confidentiality and facilitate systematic analysis.

### Researcher role

The researcher is an experienced academic specializing in teaching Turkish as a foreign language and holds a PhD in Turkish language education. This positionality enabled an in-depth and context-sensitive interpretation of the data, particularly in relation to linguistic, instructional, and cultural dimensions of the findings. At the same time, to address potential bias associated with prior expertise, the researcher engaged in ongoing reflexive practice by critically examining assumptions, grounding interpretations in participants' accounts, and documenting analytic decisions transparently. The involvement of an independent coder further supported analytical rigor and helped ensure that the findings were not solely shaped by the researcher's prior perspectives.

### Data analysis

The data were analyzed using a thematic analysis approach to identify recurring patterns and meanings across stakeholder groups. The analysis followed an iterative and systematic process involving multiple stages of coding, categorization, and theme development.

The analytical strategy sought to identify both shared and stakeholder-specific patterns across the three participant groups. Initially, interview data from students, parents, and teachers were coded separately to preserve the distinctiveness of each stakeholder perspective. Subsequently, codes and categories were compared across groups to identify patterns of convergence and divergence relevant to the research questions. This comparative process enabled the systematic integration of complementary stakeholder perspectives while preserving the distinct contributions of each group. The primary aim was to develop an integrated understanding of online Turkish language learning as a relationally mediated system while also retaining stakeholder-specific nuances where these contributed to the interpretation of the findings. Accordingly, the presentation of results emphasizes common patterns across stakeholder groups but highlights distinctive perspectives when they provide additional explanatory value.

In the first stage, all responses were read multiple times to achieve data familiarization and to gain an overall understanding of the dataset. Initial codes were then generated inductively, based on meaningful units within the data rather than predetermined categories. These codes captured participants' expressions related to meanings of learning Turkish, factors influencing the learning process, and experiences of online instruction.

In the second stage, similar codes were grouped into broader categories, and relationships between codes were examined across stakeholder groups. This process enabled the identification of patterns of convergence and divergence among students, parents, and teachers. Codes were then organized into higher-order themes corresponding to the three main dimensions of the study.

To enhance analytical transparency, the frequency of codes was calculated to indicate the relative prominence of themes within the dataset. These frequencies are reported in the findings to support the interpretation of patterns; however, they are used descriptively rather than inferentially, in line with qualitative research principles.

In addition to thematic coding, metaphorical expressions were analyzed as a complementary layer of interpretation. Metaphors were identified, categorized, and interpreted to reveal participants' underlying conceptualizations of learning and teaching Turkish.

To ensure the reliability of the coding process, an independent researcher with expertise in the field conducted a parallel coding of the data. Inter-coder agreement was calculated using the formula (agreement/[agreement + disagreement] × 100). The analysis yielded an inter-coder reliability coefficient of 95%, indicating a high level of consistency in the coding process. Discrepancies were discussed until consensus was reached, and the coding scheme was refined accordingly. Overall, the analysis was designed to ensure both analytical rigor and interpretive depth, enabling a comprehensive understanding of stakeholder experiences across contexts.

## Results

The qualitative analysis revealed a multi-layered and interconnected structure of stakeholder experiences of learning Turkish as a foreign language in online contexts. Following a systematic thematic analysis, the findings are organized around three core dimensions aligned with the research questions: (1) the construction of meanings and purposes of learning Turkish, (2) the challenges and facilitating factors shaping the learning process, and (3) the affordances and limitations of online instruction. These dimensions capture both shared and stakeholder-specific perspectives, with the analysis primarily emphasizing common patterns while drawing attention to distinctive stakeholder experiences where relevant. In the following sections, each dimension is presented with illustrative excerpts to provide a rich and nuanced account of participants' experiences.

***Theme 1. Relationally and Identity-Driven Meaning Construction of Learning*** ***Turkish***

Across all stakeholder groups, the meanings and purposes attributed to learning Turkish as a foreign language were constructed within a predominantly relational and identity-oriented framework, rather than through purely academic or instrumental considerations. The analysis revealed that Turkish is primarily positioned as a language of family connection, cultural continuity, and interpersonal belonging within transnational contexts.

Parents consistently framed Turkish learning as a family-driven necessity, emphasizing its role in sustaining intergenerational communication. As one parent expressed, “*If my child does not learn Turkish, they cannot really be part of our family conversations, especially with grandparents”* (P4). The dominant rationale centered on enabling children to communicate effectively with extended family members, particularly grandparents and relatives, and to maintain meaningful interpersonal relationships. In this sense, Turkish was constructed not as an optional educational skill but as an essential medium for sustaining familial bonds and preventing communicative disconnection across generations.

This relational orientation was reinforced by patterns of language use across contexts. Turkish was predominantly associated with home and family interactions, as well as visits to Türkiye, with limited use in school or broader social environments. Even when used outside the home, it remained largely restricted to interactions with Turkish-speaking relatives or peers. This pattern suggests that Turkish functions as a context-bound language, primarily embedded in intimate and culturally meaningful settings rather than institutional or public domains. This pattern was also reflected in students' accounts. As one stated, “*I only speak Turkish at home or when we visit Türkiye”* (S2), reinforcing the idea that Turkish is primarily embedded within family-based contexts.

Teachers' perspectives corroborated and extended these findings by situating Turkish learning within a broader framework of heritage language maintenance. They emphasized that students' engagement with Turkish is largely shaped by parental expectations and the desire to preserve linguistic and cultural ties. Teachers further emphasized the identity-related dimension, as one teacher noted, “*For many students, Turkish is not just a language—it's their connection to who they are and where they come from”* (T3). From this perspective, learning Turkish was associated with maintaining a connection to one's cultural roots, preventing language attrition, and sustaining a sense of belonging in diasporic contexts. Thus, Turkish was not only a communicative tool but also a symbolic resource linked to identity continuity and cultural affiliation.

At the same time, a secondary layer of meaning emerged in relation to language development and future-oriented utility. Some parents and students referred to improving proficiency, speaking more fluently, or potentially using Turkish in future life contexts, including living or working in Türkiye. As one student noted, “*I want to speak Turkish better in the future, maybe if we live in Türkiye”* (S6). However, these instrumental considerations remained less central compared to the dominant relational and identity-based motivations.

Students' responses further illustrated the immediacy and experiential grounding of these meanings. Their accounts focused primarily on concrete interactional contexts, such as speaking with family members at home or during visits to Türkiye. While some students expressed personal interest in improving their Turkish, their engagement was largely shaped by situational needs and family expectations, suggesting that motivation may initially emerge externally and gradually develop into more internally driven forms.

Importantly, stakeholders' willingness to continue participation in Turkish language instruction reflected the persistence of these meaning structures. Continued engagement was frequently justified in terms of improving communication, strengthening language skills, and maintaining connections to family and culture, indicating that relational and identity-based motivations also translate into sustained behavioral commitment.

Although limited in frequency, metaphorical expressions provided supplementary insights into the deeper conceptualizations of Turkish language learning. Available metaphors (e.g., learning Turkish as a “game,” a “task,” or a process requiring “understanding its logic”) reflected themes of effort, engagement, and gradual mastery. Similarly, metaphors describing Turkish lessons (e.g., “like football,” “like meeting friends,” or “like an enjoyable interaction”) highlighted the social, affective, and interactive dimensions of the learning experience. These metaphorical constructions further support the interpretation that Turkish learning is experienced as a dynamic and meaning-laden process embedded in relational and emotional contexts.

**Figure 1:**
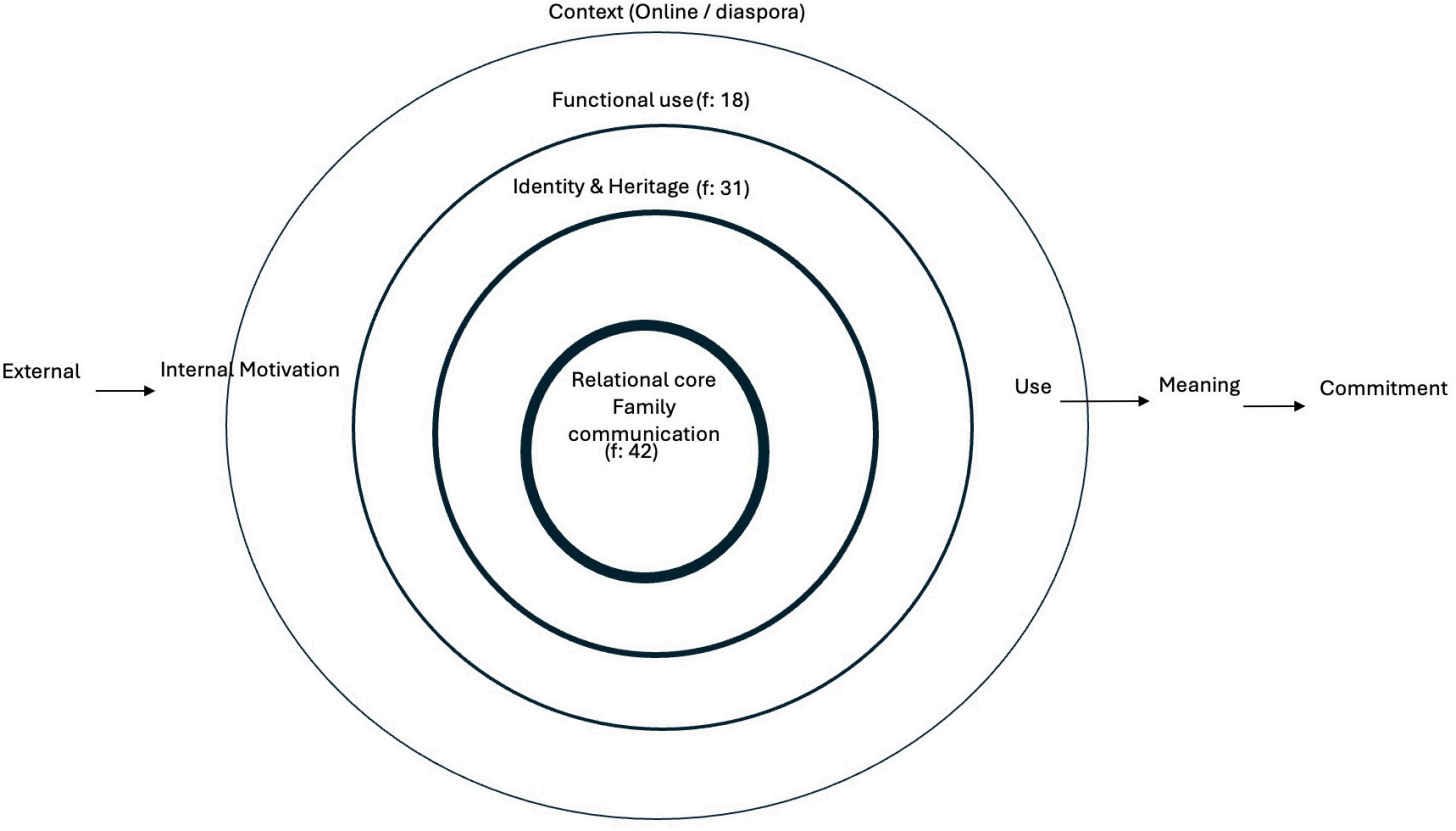
A multi-layered model of meaning construction in learning Turkish as a foreign language.

Taken together, the findings indicate that the meanings associated with learning Turkish as a foreign language are constructed through a multi-layered system in which relational (family-based) meanings constitute the core (*f* = 42), followed by identity-driven (heritage-oriented) meanings (*f* = 31), and functional (developmental and future-oriented) considerations (*f* = 18). This layered structure, illustrated in Figure 1, shows how relational meanings form the core of the system, surrounded by identity-related and functional dimensions. The arrows further represent the transition from externally driven motivation to more internalized engagement, as well as the process linking language use to meaning construction and sustained commitment. Overall, these findings suggest that motivation in this context cannot be adequately captured by traditional instrumental frameworks but instead requires a relational and context-sensitive perspective that foregrounds the role of family, identity, and everyday language use in shaping language learning processes.

***Theme 2. Factors Shaping the Learning and Teaching of Turkish in Online*** ***Contexts***

The analysis indicated that the process of learning and teaching Turkish as a foreign language in online contexts is shaped by a dynamic and interdependent system of factors operating across learner, instructional, and contextual levels. Rather than functioning as isolated influences, these factors interact to either amplify or mitigate learning challenges, thereby shaping both engagement and learning outcomes.

At the learner level, perceived difficulty and skill-specific constraints emerged as central organizing dimensions of the learning process (*f* = 28). Across stakeholder groups, Turkish was generally characterized as moderately demanding, with variation linked to individual differences and prior exposure. Difficulties were consistently concentrated in productive domains, particularly writing, vocabulary, and grammar, whereas receptive skills such as listening were perceived as relatively more accessible. Students' experiences further illustrated these challenges, with one student noting, “*Writing is the hardest part because I don't know how to form sentences”* (S5). A similar pattern was reflected in parent accounts, as one parent noted, “*My child understands, but struggles when it comes to writing or speaking”* (P3). Teachers attributed these patterns to cross-linguistic influences and the cognitive demands of lexical retrieval and sentence construction, suggesting that linguistic processing constraints systematically shape learners' engagement with the language.

A second-dimension concerns instructional mediation, particularly the role of curriculum design, materials, and teacher adaptation (*f* = 20). While some teachers described the instructional program as flexible, others identified structural limitations, including content repetition across levels, insufficient differentiation, and limited alignment with learners' developmental needs. Teachers frequently described the need to adapt instruction, as reflected in one teacher's comment: “*The materials are not always enough, so I have to change or add activities depending on the student”* (T2). In response, teachers frequently adapted or supplemented materials, indicating that instructional effectiveness is not determined by the curriculum alone but is actively constructed through teacher agency. Moreover, activity design, especially when incorporating interaction, multimodal resources, and task-based elements, emerged as a key mechanism for sustaining student engagement.

A third dimension relates to the affordances and constraints of the online learning environment (*f* = 16). Online instruction expands access and flexibility while enabling the integration of diverse digital resources. However, it simultaneously restricts opportunities for embodied interaction, spontaneous communication, and multi-sensory engagement. Stakeholders, particularly teachers, emphasized that the absence of face-to-face interaction constrains attention, engagement, and relationship-building. The limitations of online instruction were also emphasized, with one teacher stating, “*It's harder to keep students engaged because you don't have that physical presence”* (T6). At the same time, the rapid incorporation of visual and multimedia materials functions as a compensatory affordance that partially offsets these limitations.

Finally, relational dynamics operate as a cross-cutting mechanism linking these dimensions (*f* = 18). Across stakeholder groups, teacher-student and parent-mediated relational dynamics emerged as a critical factor shaping motivation, engagement, and persistence. Students' willingness to participate was closely tied to their affective connection with the teacher, while parents similarly emphasized the role of teacher characteristics in sustaining their children's engagement. Relational dynamics were particularly evident in student responses, as one student explained, “*I like the lessons because my teacher makes them fun”* (S1). This indicates that relational processes do not function as an independent factor but rather mediate how learner, instructional, and contextual conditions are experienced.

**Figure 2:**
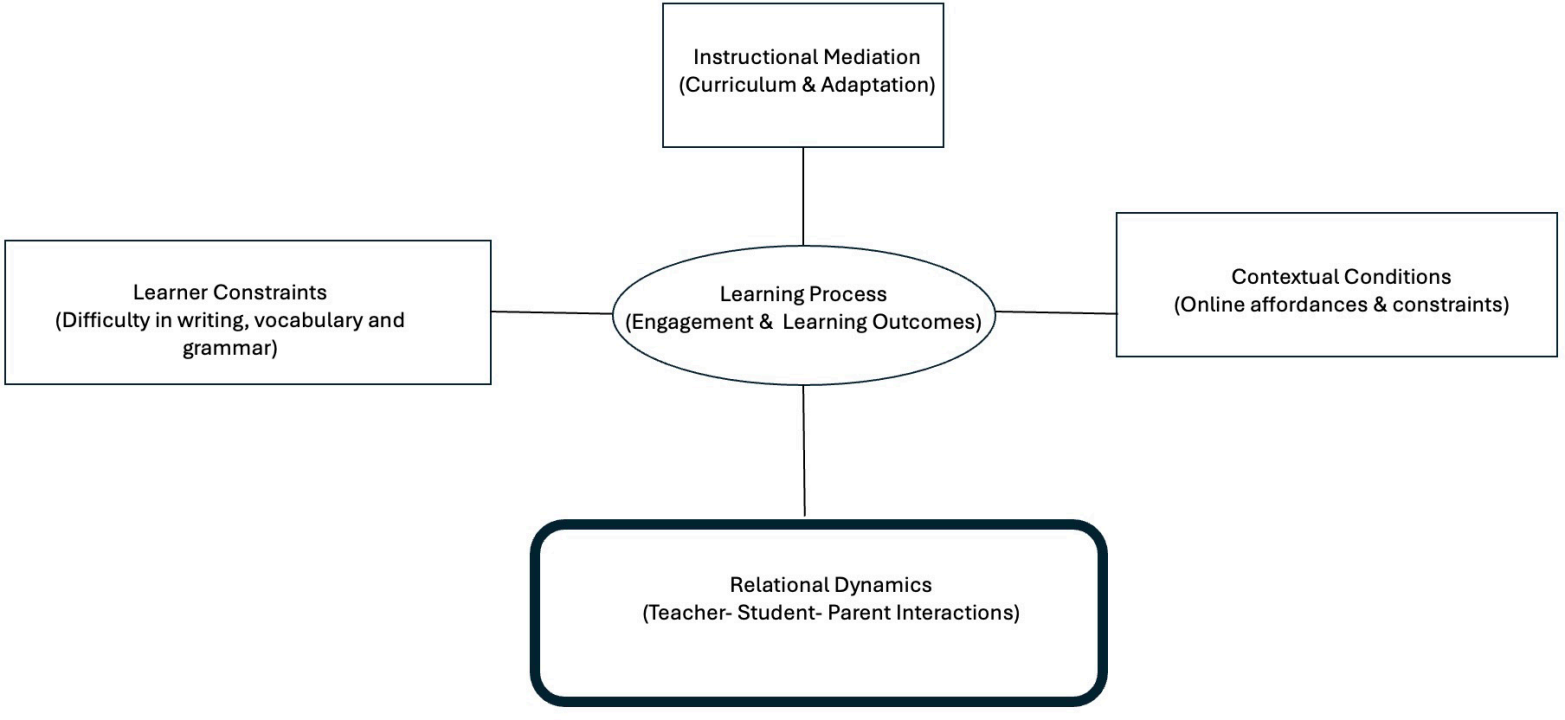
A dynamic interaction model of factors shaping online Turkish language learning.

Taken together, these findings suggest that learning and teaching Turkish in online contexts is best understood as a dynamic system in which learner-related constraints, instructional mediation, and contextual affordances interact through relational processes. Within this system, challenges and supports are not fixed properties but emerge through these interactions. As illustrated in Figure 2, this model highlights how different factors converge to shape the conditions under which learning is either facilitated or constrained. This distinction represents a key contribution of the study: while meaning construction is primarily relational, the learning process itself is shaped by the interaction between cognitive demands and instructional mediation, with relational dynamics functioning as a regulatory mechanism across these dimensions.

***Theme 3. Experiencing Online Turkish Instruction as a Relationally Mediated and*** ***Engagement-Dependent Process***

Moving beyond process-level factors, the analysis revealed that online Turkish language instruction is experienced not merely as a mode of delivery, but as a relationally mediated and engagement-dependent process shaped by the interplay between instructional design, interactional opportunities, and affective involvement. Across stakeholder groups, the quality of the learning experience was primarily defined not by the technological medium itself, but by how interaction, engagement, and relational connection were enacted within the online environment.

A defining aspect of the learning experience concerns the centrality of relational dynamics, particularly the teacher-student relationship, in shaping engagement and overall satisfaction with the learning process (*f* = 24). Both students and parents consistently emphasized the teacher as the most salient component of the online experience, frequently associating positive learning experiences with the teacher's attitude, responsiveness, and ability to establish an affective connection. The centrality of relational dynamics was clearly reflected in stakeholder accounts, as one parent stated, “*The teacher is the most important part—if my child likes the teacher, everything works”* (P6). Students' accounts highlighted enjoyment in interacting with the teacher, while parents similarly framed teacher characteristics as critical for sustaining their children's participation and motivation. This pattern indicates that, within online environments, relational quality becomes a primary determinant of how instruction is experienced.

Closely linked to this, engagement emerges as an activity-based and affectively driven process shaped by task design (*f* = 22). Across student responses, enjoyable experiences were strongly associated with interactive elements such as games, videos, and dynamic activities, which were frequently described as making the lessons fun and engaging. Students consistently linked engagement to interactive activities, with one student noting, “*I like it when we play games or watch videos - it makes learning fun”* (S7). In contrast, less preferred aspects of the learning experience were consistently linked to more demanding or effort-intensive tasks, particularly writing and homework-related activities (*f* = 14). Less engaging activities were associated with lower motivation, as reflected in one student's comment: “*I don't like writing homework - it feels boring”* (S4). This pattern suggests that engagement in online language learning is highly sensitive to task design, with interactive and multimodal activities facilitating participation, while cognitively demanding tasks may reduce motivation if not adequately supported. This dynamic was also reflected in teachers' practices, as one teacher explained, “*I use music, movement, and small games to keep students active during the lesson”* (T1).

The online environment introduces a set of conditions that both enable and constrain interaction, shaping how learning is experienced (*f* = 12). On the one hand, stakeholders highlighted the convenience of online instruction, particularly the ability to participate from home and overcome spatial and logistical barriers. On the other hand, the quality of interaction within this environment depended heavily on how instruction was structured, rather than on the medium alone. This indicates that online learning is not experienced as inherently effective or limiting, but as contingent on the ways in which interaction and engagement are pedagogically organized.

From an instructional perspective, teachers actively shape these experiential dynamics through adaptive practices (*f* = 18). Teachers reported employing a range of strategies to sustain engagement and compensate for interactional limitations, including the use of music, movement-based activities, personalized materials, and informal conversational routines. These practices reflect an adaptive and responsive approach in which teachers actively design the experiential quality of the learning environment, rather than simply delivering content.

**Figure 3:**
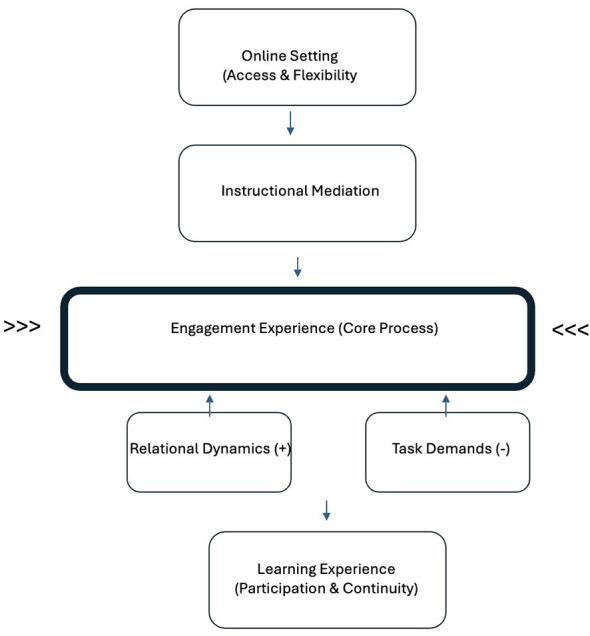
A process oriented model of experiencing online Turkish language instruction.

Overall, these patterns point to an experiential system in which relational dynamics and engagement processes jointly shape participation and learning. Rather than being defined by the technological format itself, the quality of the learning experience emerges from how interaction, activity design, and teacher - student relationships are coordinated within the online setting. As illustrated in Figure 3, these dimensions can be conceptualized as an interconnected experiential system in which relational dynamics and engagement processes jointly shape participation and learning. As such, online instruction is best understood as an interactionally constructed learning environment in which engagement and relational connection function as central drivers of participation and learning.

## Discussion

Building on the multi-stakeholder findings, the present study offers a re-conceptualization of learning Turkish as a foreign language in online contexts by showing that stakeholders' experiences are not primarily shaped by instrumental or academic goals, but are embedded in a relational and context-dependent system of meaning-making. Across students, parents, and teachers, learning Turkish emerged as a process grounded in family relationships, identity continuity, and affective connections rather than as a purely skill-based activity. Beyond this, the findings indicate that learning operates as a dynamic system shaped by the interaction of learner-related constraints, instructional mediation, and contextual affordances, with relational dynamics regulating how these elements are experienced (Hiver et al., 2021; Larsen-Freeman, 2018). Importantly, online instruction was not perceived as a neutral technological medium, but as an interactionally constructed environment in which engagement and relational connection shape participation and learning (Hodges et al., 2020; Lai, 2019). Collectively, these results challenge individualistic and instrumentally driven perspectives on language learning, positioning it instead as a relationally mediated and context-sensitive process. In line with the three research questions, the discussion is organized around the interconnected dimensions of meaning construction, learning processes, and experiences of online instruction before integrating these findings into the proposed relationally mediated learning system.

### Meaning construction and relational foundations of Turkish language learning

Focusing more specifically on the meanings attributed to language learning, the findings indicate that these meanings are predominantly relational and identity-based, extending existing research on heritage language learning and family language policy. Prior studies have highlighted the role of family expectations and intergenerational communication in sustaining heritage language engagement (Curdt-Christiansen et al., 2023; Li et al., 2026). However, the present findings suggest that these relational dynamics do not simply act as external motivators but form the core meaning system through which language learning is experienced. From this perspective, motivation is better understood not as an individual cognitive construct, but as a socially embedded process shaped by everyday family practices (Dunn and Iwaniec, 2022; Kim, 2023). Furthermore, the layered structure identified in this study, where relational meanings constitute the core, surrounded by identity-related and functional dimensions, points to a developmental process in which externally driven participation may become internalized as part of learners' sense of self. In this way, the findings extend traditional distinctions between instrumental and integrative motivation by suggesting that, in transnational and heritage language contexts, motivation is more accurately conceptualized as a relational ecology shaped by identity, belonging, and family interaction (Boo et al., 2015).

### Learning processes as relational and context-dependent systems

Extending this meaning-centered perspective to the learning process itself, the findings indicate that learning and teaching Turkish in online contexts are not adequately explained through linear or single-factor models but are better understood as a dynamic system shaped by the interplay of learner characteristics, instructional mediation, and contextual conditions. This view is consistent with process-oriented and ecological approaches, which conceptualize learning outcomes as emerging from interactions among individual, instructional, and environmental factors rather than isolated variables (Kusyk et al., 2025; Larsen-Freeman, 2018). In this study, perceived difficulty, particularly in productive skills such as writing and speaking, did not reflect a fixed learner deficit, but emerged from the interaction between cognitive demands, linguistic complexity, and the level of instructional support provided. This interpretation aligns with research on cognitive load and second language processing, suggesting that task demands and scaffolding jointly shape learners' capacity to engage with complex language structures. Importantly, the findings highlight the central role of instructional mediation, especially through teacher adaptation and task design, in transforming potential constraints into manageable challenges (Hiver et al., 2021). Within this framework, relational processes function as a regulatory mechanism that shapes how learners experience difficulty, engage with tasks, and sustain participation. Accordingly, learning can be understood not as the accumulation of discrete skills, but as an emergent process arising from the coordinated interaction of cognitive, instructional, and relational dimensions. This shifts the interpretation of learning difficulty from an individual deficit to a system-level phenomenon of variability in learner engagement and performance, particularly in linguistically and contextually complex online environments.

### Experiencing online Turkish instruction through engagement and relationships

Shifting from process-level dynamics to the experiential dimension of learning, the findings provide important insights into online language learning environments by showing that the quality of the learning experience is shaped less by the technological medium itself and more by how interaction and engagement are pedagogically organized (Hodges et al., 2020). This challenges technologically deterministic views of online education, which often portray digital environments as inherently enabling or constraining learning outcomes. Instead, the results align with engagement-oriented perspectives, suggesting that meaningful learning depends on learners' active participation in socially mediated and affectively engaging activities (Bolliger and Martin, 2018; Hiver et al., 2021). In this study, engagement emerged as highly sensitive to task design: interactive and multimodal activities enhanced participation, whereas cognitively demanding tasks, such as writing, reduced motivation when insufficiently supported. A key finding is the central role of the teacher-student relationship in shaping how online instruction is experienced (Hiver et al., 2021; Hodges et al., 2020). Rather than functioning as a secondary support, relational quality appears to be a primary condition for sustaining participation. Teachers' adaptive practices, including the use of personalized materials and interactive routines, further illustrate that online learning environments are actively constructed rather than passively experienced. Overall, these patterns indicate that online language learning is best understood as a pedagogically shaped process in which engagement and relational connection function as key drivers of participation.

### Toward a relationally mediated learning system

Synthesizing these interconnected findings, the study points to the need for a more integrated conceptualization of language learning that moves beyond fragmented accounts of motivation, instruction, and engagement (Larsen-Freeman, 2018). Based on the multi-stakeholder evidence, this study proposes a *relationally mediated learning system* in which meaning construction, learning processes, and engagement are interconnected. At the core of this system is relational meaning, grounded in family interaction and identity continuity, which provides the primary motivational basis for participation. Within this framework, learning outcomes emerge from system-level interactions rather than isolated learner characteristics (Kusyk et al., 2025). Engagement functions as a linking mechanism that translates meaning into active participation, shaped by the design of learning activities. Relational dynamics further organize how learners experience difficulty, respond to instruction, and sustain participation over time. This model reconceptualizes language learning as a socially embedded and dynamically regulated process rather than an individual, skill-based achievement. By integrating these dimensions within a single framework, the study shifts the focus from individual, skill-based accounts toward a more holistic understanding of learning as an emergent system.

### Theoretical contributions

Building on this integrative framework, the present study offers several important theoretical contributions to the literature on language learning, particularly in online and transnational heritage language contexts. First, it advances motivation theory by reconceptualizing motivation not as an individual and predominantly instrumental construct, but as a relationally embedded and context-dependent process grounded in family interaction, identity continuity, and everyday language use (Dunn and Iwaniec, 2022; Kim, 2023). By introducing the notion of a *relational ecology of motivation*, the study extends traditional distinctions between instrumental and integrative orientations, suggesting that motivation in transnational contexts emerges from socially situated practices. Second, the study reframes learning difficulty not as an individual deficit, but as an emergent property of system-level interactions. This perspective moves beyond deficit-oriented interpretations of learner difficulty and instead positions learning outcomes as emergent from these interactions. Third, the study contributes to research on online learning by challenging technologically deterministic perspectives and showing that the quality of instruction depends on how engagement and relational connection are pedagogically enacted (Hodges et al., 2020). In this regard, the findings highlight the central role of teacher-student relationships and task design in shaping meaningful learning experiences. Taken together, these contributions culminate in the proposal of a *relationally mediated learning system*, an integrative framework that brings together meaning construction, learning processes, and engagement within a single model. This framework offers a theoretically grounded lens for understanding language learning in complex, transnational, and digitally mediated contexts.

Overall, the principal contribution of this study lies in demonstrating that online Turkish language learning in transnational contexts is shaped by the interaction of family-based meanings, identity continuity, teacher–student relationships, and engagement processes rather than by isolated cognitive or technological factors. By integrating these dimensions within a relationally mediated learning system, the study offers a focused and context-sensitive framework for understanding how language learning is experienced and sustained across online L2 and heritage language settings. Rather than proposing a universal model, the framework provides a relational perspective that may inform future research and practice in digitally mediated language education.

### Practical implications

Translating these theoretical insights into practice, the findings of this study offer several implications for multiple stakeholders involved in online heritage language education. For teachers, effective instruction in online contexts depends less on the technological tools themselves and more on how learning experiences are designed (Bolliger and Martin, 2018). Rather than prioritizing content coverage alone, teachers should create interactive and meaningful learning environments by incorporating multimodal activities, authentic communication opportunities, and flexible adaptations that respond to individual learner needs. Importantly, cognitively demanding tasks, such as writing, should not be avoided but carefully scaffolded through structured support and gradual progression to sustain engagement.

For families, the findings highlight that language learning extends beyond formal instruction and is embedded in everyday practices. Regular use of Turkish in meaningful interactions, such as conversations, shared activities, and communication with extended family, can play a central role in supporting both motivation and language development. In this sense, parents function not only as supporters but as active contributors to the linguistic environment that sustains learning. This underscores the need to conceptualize families as integral components of the instructional system, rather than as external supporters, as language development is continuously shaped through everyday interactional practices (Curdt-Christiansen et al., 2023; Li et al., 2026).

Finally, for program developers and policymakers, the results suggest that standardized and one-size-fits-all curricula may be insufficient for addressing the diverse needs of learners in transnational contexts. Instructional programs should therefore allow for flexibility, teacher adaptation, and the inclusion of culturally relevant content. In addition, teacher training should emphasize not only pedagogical knowledge but also strategies for fostering connection and maintaining engagement in online environments. This emphasis is consistent with recent work highlighting the growing importance of teacher-related factors and instructional beliefs in shaping effective educational practices across diverse learning contexts (Wang et al., 2024). At its core, effective online language learning is not simply a matter of content delivery, but of designing meaningful and context-sensitive experiences that connect language use with learners' everyday lives.

### Limitations and future research

While the study provides important insights, several limitations should be considered when interpreting the findings, which also point to directions for future research. First, the study relied on a relatively small and purposively selected sample which, while appropriate for in-depth qualitative exploration and strengthened by the inclusion of multiple stakeholder perspectives, may limit the transferability of the findings to other contexts; future studies with larger and more diverse samples would allow for broader examination across different learner populations and instructional settings. Second, although data were collected through semi-structured interviews, the online format may have constrained the depth and spontaneity of interaction to some extent, particularly in relation to non-verbal communication and natural conversational flow; future research could incorporate face-to-face interviews, observations, or interactional analyses to capture more nuanced and real-time meaning-making processes. Third, in the case of younger participants, interviews were conducted with parental support when necessary, which introduces the possibility of mediation or unintentional influence; although efforts were made to preserve the authenticity of student responses, future studies could address this limitation by employing fully independent, age-appropriate interview techniques or digital self-recording methods. Finally, the cross-sectional design limits the ability to capture developmental changes in meaning construction, engagement, and learning processes over time, highlighting the need for longitudinal research that examines how these dimensions evolve across different stages of language development. Building on these considerations, future research may also benefit from multi-method designs that integrate qualitative and quantitative approaches to provide a more comprehensive understanding of how relational, instructional, and engagement-related processes influence learning outcomes. In addition, comparative studies across different linguistic and cultural contexts would be valuable for testing the generalizability of the proposed relationally mediated learning system, while intervention-based and design-oriented research could examine how relational and engagement-focused instructional practices can be systematically implemented and evaluated in real-world settings.

## Data Availability

The original contributions presented in the study are included in the article/supplementary material, further inquiries can be directed to the corresponding author/s.
